# Professional Ethics

**Published:** 1859-06

**Authors:** J. Taft


### PROFESSIONAL ETHICS.

BY J. TAFT.

THERE are some rules of conduct which dentists, as members of an honorable profession, should exhibit toward each other. There are some things that are obviously binding upon each and every member of the profession toward all other members. There are some rules of conduct that will be modified by circumstances—things that in one case should control, while in others, they may with perfect propriety be wholly disregarded. There are, then, some things that should be observed and exhibited to all honorable members of the profession, at all times, and under all circumstances.

First, a warm interest and fraternal feeling toward each other should characterize every one. This is a natural principle, and springs forth with a spontaneous and vigorous growth, when not checked by ambition, jealousy, love of money, or conflicting interests. Those engaged in the same enterprise, laboring in the same cause, or subject to difficulties and trials of like character, will always have a common sympathy; they have interests, trials, and difficulties into which none can fully enter but those engaged. This principle, in all the avocations and occupations of life, goes to make up the different departments and distinct associations. A special sympathy, predicated upon this, is an innate principle. Persons engaged in similar avocations, are ordinarily more interested in each other's society, than those of wholly diverse pursuits, because they have a common stock or fund, and each may have a desire to know more, and also to return an equivalent for what he receives. A fraternal professional feeling will be manifested in various ways; and first, by a freedom of communication in that which pertains to the practice of the profession. Withholding a knowledge of the modes of practice, or anything that would materially affect it, is wholly incompatible with the genius of a liberal and honorable profession. The dentist should be willing to *receive*, and so far as he can, render an equivalent in the same way, and, in addition, make a free acknowledgement of the same, if circumstances require it. The true principle and feeling would say, I give what little I have, hoping that by a different and better culture, others will greatly increase it. In the present state of the dental profession, a free interchange of opinion is profitable for all. The question whether the truly professional man should have modes of practice, whether they originated with him or others—it matters not—which he may with propriety withhold from the profession, is one, about which there has been some diversity of opinion; this has reference, of course, only to those things which have or may have real merit. Experience proves the fact pretty fully, that it is as a general thing, the best policy to withhold nothing from the profession. This is particularly true of those who make the practice of their profession their avocation, and rely upon it for their resources. Every practitioner is constantly receiving fresh supplies, in regard to modes of practice from the profession at large. No one will deny that the secret, or nostrum holder, if he makes that his business and looks to it for his resources, has a perfect right to sell whenever and wherever he can, then, he does not receive an equivalent in valuable particulars of practice, and if he did, they would be of no value to him, inasmuch as he could not use them, and it is but just that he should have an equivalent in money for his secret process, if it is a good one. It is certainly better, under all circumstances, where a man is dependent upon his profession for fresh supplies in regard to modes of practice, to be liberal, even if he should give more than he receives; but in the present stage of the dental profession, that is impossible. Again, under all circumstances, we should concede to every member of the profession, full credit and acknowledgement, for all that he has; this has not always been done, but time is certainly making an improvement in this respect. Private personal feelings should never cause any one to do injustice to the attainments and abilities of a professional brother. This is sometimes done in an indirect, but very efficient manner. There are some in the dental profession, as in other professions, who, when anything new is presented to them, affirm, or insinuate, that they had accomplished the same thing long before, if not in exactly the same method, by one very similar. Ordinarily when such difference comes to be investigated closely, it is found to be much greater than would be inferred from the remarks; and this is generally true of the thing accomplished, as well as of the method of accomplishing it. Operators in the same vicinity should, as nearly as possible, adopt a uniform rate of fees; this, justice to all parties demands. No dentist can, with justice to himself, make his fee rates less than those of an honorable competitor, unless his operations are inferior in character; then his fees should certainly be lower than those who do good work. Generally, under-bidding is an evidence that the operator has not the ability or inclination to make better work. If he feels that he can not perform as good operations as his neighbors, then his charges should be less than theirs. A dentist's fees may be regarded as a very just criterion of the character of his work. Operators in the same locality will harmonize better, can elevate the character of the operations at pleasure, and do quite as much business, and be better remunerated for what is done, and give more satisfaction to the patients, where fee bills are uniform. When it is otherwise, patients are always suspicious, or dissatisfied. Fearful on the one hand, lest they are imposed upon in the character of the work, and on the other lest they are imposed upon by the fee charged, and thus they are kept in a state of vacillation, not knowing whether there is justice in the work performed, or in the fee charged. He who proposes to perform an operation for less than his neighbor has proposed, when he is aware of the fact, admits that he intends to make an inferior piece of work, or that his neighbor is taking the advantage of his patient, or, in other words, defrauding him.

